# Nomogram predicting pulmonary metastasis of hepatocellular carcinoma after liver transplantation

**DOI:** 10.18632/oncotarget.23418

**Published:** 2017-12-19

**Authors:** Li-Feng Huang, Ping Wan, Dong-Wei Xu, Seogsong Jeong, Ming-Xuan Feng, Jian-Jun Zhang, Qiang Xia

**Affiliations:** ^1^ Department of Hepatic Surgery, Ren Ji Hospital, School of Medicine, Shanghai Jiao Tong University, Shanghai 200127, China

**Keywords:** nomogram, liver transplantation, hepatocellular carcinoma, prognosis, pulmonary metastasis

## Abstract

A novel prognostic nomogram predicting post-transplant pulmonary metastasis was established with a primary cohort of 308 HCC patients who received liver transplantation between 2007 and 2011 at Ren Ji Hospital. The C-indexes for predicting pulmonary metastasis was 0.85. The calibration curves fitted well between the predicted and actual outcomes. The decision curve analysis indicated that our nomogram was the optimal decision-making strategy for PM prediction compared to Milan, University of California San Franscisco, and up-to-seven criteria. These results were further validated by data from 103 patients who underwent liver transplantation between 2011 and 2012 at the same institution. In conclusion, our nomogram could be used as an effective tool to predict PM after liver transplantation.

## INTRODUCTION

Hepatocellular carcinoma (HCC) is the sixth most common cancer and a primary cause of cancer-related mortality [1]. Liver transplantation (LT) is an optimal therapy for liver cancer, because it can radically remove the primary tumor and treat underlying liver diseases, such as primary biliary cirrhosis (PBC) and hepatitis B virus (HBV)-associated cirrhosis. However, tumor recurrence after LT is frequently extrahepatic and could significantly influence the survival outcomes of patients. Pulmonary metastasis (PM) accounts for nearly half of all extrahepatic metastases after LT [2–4].

PM following curative LT for HCC indicates a poor prognosis. However, postoperative management of PM could significantly prolong patient survival. Several institutions have suggested that pneumonectomy for PM in patients with HCC following LT may improve long-term survival, but this procedure must be performed delicately in highly-selected patients, especially patients with maximum tumor size of the pulmonary metastasis < 33 cm [5–7]. Radiofrequency ablation (RFA) for lung metastasis of HCC was recently reported as a minimally invasive treatment with an effectiveness rate of 92% and improved survival rate [8, 9]. Jang et al. demonstrated that the application of conformal radiotherapy and tomotherapy, including helical tomotherapy, increased the partial/complete remission rate of PM [10]. Despite advances in the treatment of PM, approaches for accurate prediction of PM incidence after LT remain insufficient. Accurate identification of high-risk patients may lead to early detection of PM and prolong overall survival.

Precise prediction of postoperative PM is challenging due to the multitude of incidence factors. Existing clinical staging systems can be applied to predict PM development [11–13], but were not developed for this purpose. The nomogram is an established and accurate model for clinical prediction [14–16]. In this study, we developed a nomogram to predict the postoperative development of PM in patients with HCC.

## RESULTS

### Clinicopathological characteristics

The clinicopathological characteristics of primary and validation cohorts are summarized in Tables 1 and 2. The majority of the patients were male (86.1%), hepatitis B surface antigen-positive (89.8%), and single nodule (72.7%). No statistical difference was found in the clinicopathological characteristics of the cohorts.

**Table 1:** Baseline patients characteristics

Variables	Primary cohort		Validation cohort	
	No. of patients	%	No. of patients	%
**No. of patients**	308	100	103	100
**Age, years**				
Median Range	51 28-72		50 30-69	
**Sex**				
Male Female	262 46	85.1 14.9	92 11	89.3 10.7
**HBsAg**				
Negative Positive	27 281	8.8 91.2	15 88	14.6 85.4
**HCV-Ab**				
Negative Positive	300 8	97.4 2.6	99 4	96.1 3.9
**PT, second**				
Median Range	14.5 10.6-35.7		14.2 9.4-31.7	
**ALB, g/L**				
Median Range	35.6 16-63.8		35.9 21-55	
**ALT, U/L**				
Median Range	42.2 10-217		50.8 9-295	
**AFP, ng/mL**				
Median Range	654.4 1-4800		794.3 1.3-4789	
**CA19-9, U/mL**				
Median Range	58.7 0.1-568		46.1 0.8-423	
**MELD score**				
Median Range	12.6 6.4-36.1		10.8 6.4-29.1	
**Cirrhosis**				
No Yes	45 263	14.6 85.4	9 94	8.7 91.3
**Surgical intervention**				
DDLT LDLT	266 42	86.4 13.6	91 12	88.3 11.7

Abbreviations: AFP, alpha-fetoprotein; ALB, albumin; ALT, alanine aminotransferase; CA19-9, carbohydrate antigen 19-9; DDLT, deceased-donor liver transplantation; HBsAg, hepatitis B surface antigen; HCV, hepatitis C virus; LDLT, living-donor liver transplantation; MELD, model for end-stage liver disease; PT, prothrombin time.

**Table 2:** Pathological characteristics of tumor

Variables	Primary cohort		Validation cohort	
	No. of patients	%	No. of patients	%
**No. of patients**	308	100	103	100
**Tumor size, cm**				
Median Range	5.7 1-30		5.1 1-18	
**Nodules**				
Single Multiple	229 79	74.4 25.6	70 33	68.0 32.0
**Capsule**				
Incomplete Complete	260 48	84.4 15.6	87 16	84.5 15.5
**Vascular invasion**				
None Microvascular invasion Macrovascular invasion	240 30 38	77.9 9.7 12.3	77 11 15	74.8 10.7 14.6
**Differentiation**				
I-II III-IV	252 56	81.8 18.2	82 21	79.6 20.4
**Mlian criteria**				
Within^†^ Beyond	185 123	60.1 39.9	58 45	56.3 43.7

^†^Includes tumor within and downstaged to Milan criteria before Liver transplantation. Microvascular invasion indicates number of patients with microvascular invasion, but without macrovascular invasion. Macrovascular invasion includes both the patients with or without microvascular invasion.

### Clinical outcome in primary cohort

The median follow-up of the primary cohort was 48 months (range, 1-97) and the median time to the development of PM was 23 month (range, 1-94). The postoperative overall survival (OS) and tumor recurrence rates were 71.5%, 55.9%, 52.2%, and 33.1%, 40.4%, 42.7% at 1, 3, and 5 years, respectively. Among patients with postoperative recurrence or metastasis, 72 patients were diagnosed with PM before or simultaneously with other recurrences: PM only (n=35), PM with intrahepatic recurrence (n=13), PM with abdomen/pelvis metastasis (n=8), PM with adrenal gland metastasis (n=2), PM with lymph gland metastasis (n=5), PM with bone metastasis (n=7), or PM with brain metastasis (n=2). In addition, 21 patients had intrahepatic recurrence only. Extrapulmonary metastases were demonstrated in 23 patients: abdomen/pelvis (n=7), adrenal gland (n=2), lymph gland (n=3), bone (n=10), and brain (n=1). Intrahepatic recurrence combined with distant metastases (except lung) were found in 16 patients.

### Construction of nomogram

**Figure 1:**
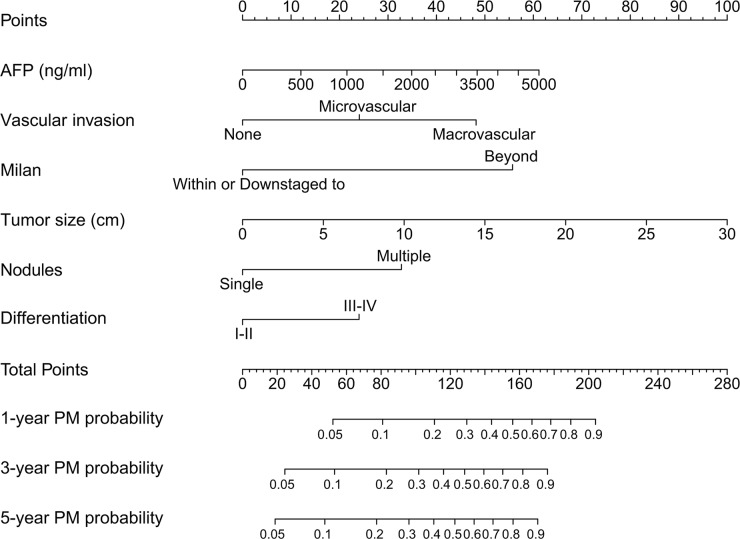
Nomogram for PM prediction after LT AFP, serum α-fetoprotein level before LT; Milan, tumor within or downstaged to the Milan criteria before LT; Differentiation, tumor histologic differentiation.

**Figure 2:**
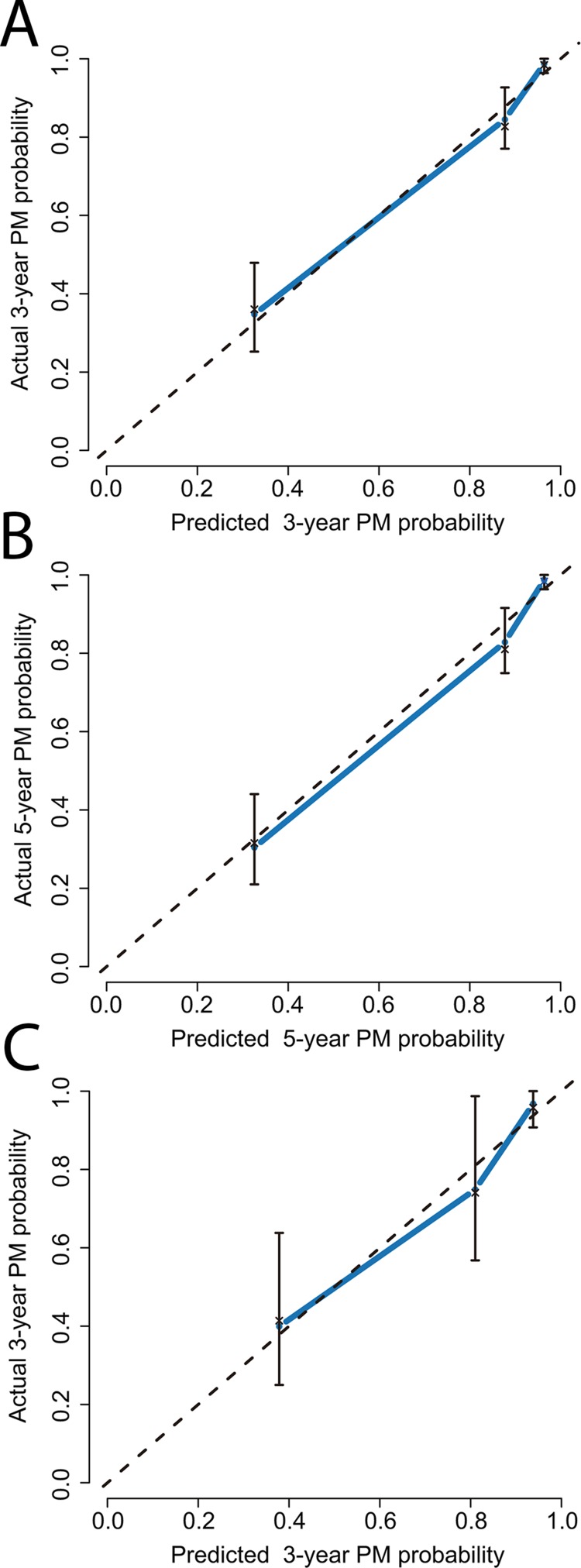
The calibration curve analysis for PM prediction **(A)** 3 years after LT in the primary cohort; **(B)** 5 years after LT in primary cohort; **(C)** 3 years after LT in the validation cohort.

Univariate and multivariate analyses demonstrated that preoperative serum AFP, vascular invasion, within or downstaged to Milan criteria, tumor diameter, tumor nodules, and tumor differentiation were found to be independent factors that influenced the incidence of PM (Table 3). These six variables were included in the nomogram (Figure 1). The model was accurate with a concordance index (C-index) of 0.85. The calibration plot indicated a strong correlation between actual PM probability and predicted PM probability (Figure 2A and 2B).

**Table 3:** Independent risk factors predicting PM in the primary cohort

Variables	Univariate analysis			Multivariate analysis		
	HR	95% CI	*P-*value	HR	95% CI	*P*-value
Age, years	0.97	0.94-0.99	**0.006**			NA
Sex: Male vs. Female	2.32	0.94-5.75	0.070			NA
HBsAg: Positive vs. Negative	1.22	0.49-3.02	0.673			NA
PT, second	0.94	0.88-1.01	0.089			NA
ALB, g/L	1.02	0.99-1.06	0.192			NA
ALT, U/L	1.00	0.99-1.01	0.338			NA
AFP, ng/mL	1.00	1.00-1.01	**<0.001**	1.00	1.00-1.01	**0.001**
CA19-9, U/mL	1.01	1.01-1.04	0.074			NA
MELD score	0.99	0.94-1.03	0.581			NA
Cirrhosis: Yes vs. No	1.20	0.60-2.42	0.606			NA
Tumor size, cm	1.15	1.12-1.19	**<0.001**	1.08	1.02-1.13	**0.006**
Nodules: Multiple vs. Single	1.53	1.18-1.98	**0.001**	1.42	1.05-1.92	**0.021**
Capsule: Complete vs. Incomplete	0.28	0.10-0.76	**0.013**			NA
Vascular invasion	2.80	2.18-3.61	**<0.001**	1.90	1.38-2.62	**<0.001**
Differentiation: III-IV vs. I-II	4.92	3.04-7.95	**<0.001**	1.81	1.08-3.04	**0.024**
Surgical intervention: DDLT vs LDLT	2.08	0.90-4.79	**0.087**			NA
Milan: Within^†^ vs. Beyond	0.07	0.04-1.41	**<0.001**	0.22	0.10-0.49	**<0.001**

Abbreviations: AFP, alpha-fetoprotein; ALB, albumin; ALT, alanine aminotransferase; CA19-9, carbohydrate antigen 19-9; CI, confidence interval; DDLT, deceased-donor liver transplantation; HBsAg, hepatitis B surface antigen; HR, hazard ratio; LDLT, living-donor liver transplantation; MELD, model for end-stage liver disease; PT, prothrombin time.

^†^Includes tumor within and downstaged to Milan criteria before Liver transplantation.

Note: Univariate and Multivariate analysis, competing risk regression model. Bold values indicate P<0.05.

### Validation of nomogram

In the validation cohort, the median follow-up was 33 months (range, 1-44). The median time to the detection of PM was 21 months (range, 1-40). The OS and recurrence rates at 1 and 3-years were 72.8%, 57.6%, and 32.4%, 46.5%, respectively. Forty-eight patients had recurrence or metastasis after LT: PM (either before or with concomitant metastasis, n = 25), intrahepatic recurrence only (n = 9), extrapulmonary metastases only (n = 10), and intrahepatic recurrence combined with distant metastases excluding lung (n = 4). Among the 25 patients with postoperative PM, 11 patients presented with PM only, 5 patients presented PM with concomitant intrahepatic recurrence, 2 patients presented PM with concomitant abdomen/pelvis metastasis, 3 patients presented PM with concomitant lymph gland metastasis, and 4 patients presented PM with concomitant bone metastasis. The nomogram showed a C-index of 0.83 with a well-fitted calibration plot (Figure 2C).

### Comparison of nomogram

**Figure 3:**
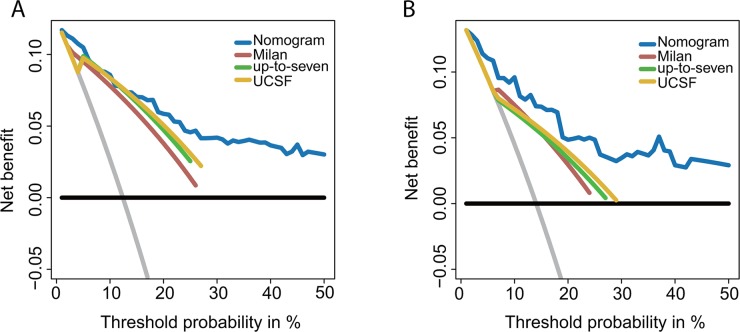
DCA of nomogram and conventional criteria The net benefits and the threshold probabilities are presented on Y-axis and X-axis, respectively. **(A)** Comparison within the primary cohort; **(B)** comparison within the validation cohort; horizontal black line, PM did not occur in any patients; gray line, all the patients developed PM.

In our study, the nomogram demonstrates the highest curve compared to Milan, UCSF, and up-to seven criteria, revealing that it is the optimal decision-making strategy for PM prediction [11–13] (Figure 3).

## DISCUSSION

Tumor recurrence is the representative indication of poor prognosis in patients with HCC. The lung is the most common site of metastasis after LT. Successful treatment of PM by pneumonectomy and adjuvant therapy could prolong long-term survival in HCC patients [5, 7, 17]. Our nomogram was developed from comprehensive clinicopathological characteristics based on serological examinations to accurately predict the development of PM after LT. Previous reports utilizing clinical stage systems focused on the tumor diameter and number, which are influential factors for predicting tumor recurrence [12, 13]. In addition to these two parameters, we included vascular invasion, histologic differentiation, the serum level of AFP, and tumor-downstaging to Milan criteria in our nomogram. Vascular invasion indicates that the malignancy has invaded blood vessels and represents the possibility of distant metastases. A majority of transplant centers included vascular invasion (macro- and microvascular) in their models to estimate OS or recurrence after LT [11, 18–19]. Histological differentiation is another critical factor in the prognosis of patients who received LT and considerable attention has been paid to its clinical application [20, 21]. Serum level of AFP is pivotal to the invasive/metastatic capacity of HCC cells and is directly associated with prognosis [22]. A recent study by Li et al. indicated that AFP levels were an independent factor that effectively predicted the occurrence of PM after hepatic resection [23]. Another study by Duvoux et al. demonstrated that the prediction of tumor recurrence was improved by including AFP level in the estimation model [24]. HCC exceeding the Milan criteria can now be downstaged before surgical operation. Improved tumor-free survival and OS has been reported in patients who received preoperative downstaging treatment and was comparable to survival outcomes of those within the Milan criteria [25, 26]. The presence of downstaging treatment was added to our nomogram.

Prediction models have become ubiquitous for OS and tumor recurrence prediction [15, 27, 28]. The nomogram is a statistical model designed to maximize predictive accuracy and is more effective than the other models for estimating multiple clinical end points [29, 30]. Our nomogram accurately predicted PM development (C-index = 0.85). DCA was used to evaluate the predictive performance of our nomogram because it is useful for assessing and comparing models that contain clinical consequences [31]. According to DCA, the nomogram is the optimal decision-making strategy compared to three well-known staging systems.

In the diagnostic procedures of PM, we could not exclude the probability of primary lung malignancy. However, the incidence of primary lung cancer in HCC patients showed a rate of less than 1% [32]. According to this incidence, the number of patients who had primary lung cancer should be no more than 2. In addition, the diagnosis of PM was based on the results from imaging studies and AFP serum level, which were sensitive enough to detect most primary lung cancers [23]. So, it is our stringent diagnostic criteria and the low incidence of primary pulmonary cancer supported the reliability of our results.

Several limitations existed: 1) as a single institutional study, large-scale randomized controlled studies are necessary to further validate this model before clinical approval; 2) the majority of our patients were HBV-associated (89.8%) or cirrhotic (86.9%), so the model might not be applicable to patients with other disease distributions; and 3) PM after LT was diagnosed by radiological imaging and serological examination. Although the incidence of primary lung cancer in patients with HCC is extremely low, the possibility could not be excluded entirely.

Our nomogram accurately predicted the development of PM in patients with HCC who received LT. Our results could be utilized to determine the risk index of PM in HCC patients for intensified surveillance and postoperative treatment.

## MATERIALS AND METHODS

### Patients

Between January 2007 and January 2011, patients with HCC who underwent LT at the Ren Ji Hospital (Shanghai, China) composed the primary cohort. The patients who received LT for HCC from February 2007 to December 2011 formed the validation cohort. The data of both cohorts were collected prospectively and analyzed retrospectively. All the surgical procedures and postoperative immunosuppressive treatments were performed as described in our previous study [33]. Transplantation and organ donation in the study was carried out strictly in accordance with the regulations of the Shanghai Organ Transplant Committee and the Declaration of Helsinki. Ethical approval was obtained from the Committee of Ethics at Ren Ji Hospital. Informed consent was acquired before LT. All cadaveric donor tissues involved in the study were obtained from brain-dead or no-heart beating donors. This study was censored in March 2015.

In the primary cohort, we excluded 23 patients for the following reasons: combination with other tumors (n=7; 4 confirmed before and 3 after LT), possible metastatic disease before LT (n=8), possible preoperative pulmonary lesions (n=3), perioperative mortality (n=3), and incomplete data (n=2). A total of 308 patients met the eligibility criteria and comprised the primary cohort. In the validation cohort, 12 patients were excluded according to the following reasons: combination with other tumors (n=5; 3 confirmed before and 2 after LT), possible metastatic disease before LT (n=4), potential preoperative pulmonary lesions (n=1), and perioperative mortality (n=2). The validation cohort was composed of 103 patients.

### Data collection

Routine serological examinations of hepatitis B and C immunology, liver function, prothrombin time (PT), carbohydrate antigen 19-9 (CA19-9), and α-fetoprotein (AFP) were performed. Additional examinations included chest radiograph, abdominal ultrasound, magnetic resonance imaging (MRI), and contrast-enhanced computed tomography (CT). Patients with suspected distant metastases received further examination by positron emission tomography (PET). The severity of preoperative disease was evaluated by using the clinical manifestation, radiograph, and serological biomarkers. Preoperative downstaging treatment for HCC included radiofrequency ablation (RFA), percutaneous ethanol injection (PEI), and transcatheter arterial chemoembolization (TACE).

### Follow-up

Patients were observed monthly in the first 6 months after LT and once every 3 to 6 months thereafter. Routine abdominal ultrasound and chest radiography were performed at each follow-up visit. Other examinations included serum AFP, CA19-9, and liver function tests. When tumor recurrence was suspected, a chest CT scan and contrast-enhanced CT/MRI of the whole abdomen were performed. PM was diagnosed by the following criteria: (a) detection of growing lesions from dynamic chest CT scan and (b) elevated serum AFP level. Other pulmonary lesions were distinguished by a bronchial perfusate test and cytological examination of sputum. Patients who were diagnosed with PM before or simultaneously with extrapulmonary metastases were defined as our subjects.

### Management of pulmonary metastasis

Patients with solitary pulmonary metastasis were referred to thoracic surgeons for consideration of surgical resection. The criteria for surgical resection included the feasibility of complete removal of the lung lesion and sufficient pulmonary function after surgery. In PM patients with concurrent intrahepatic or other extrahepatic recurrence, lung resection performed after complete control of extrapulmonary metastasis. Systemic treatment with sorafenib or conventional chemotherapeutic (doxorubicin and cisplatin) was administered in patients with multifocal disease and unresectable lesions. All available treatment modalities including surgical resection, transarterial chemoembolization, radiofrequency ablation, radiotherapy, and chemotherapy were applied to the extrapulmonary metastases.

### Statistical analysis

The statistical analysis was performed using R version 3.3.1 for Windows (http://www.r-project.org/) and SPSS 15.0 (SPSS, Chicago, IL). The endpoints of this study were the overall survival (OS), time to recurrence, and the time to PM. Treating extrapulmonary metastasis and death before PM as competing events, we performed a competing risk survival analysis to identify independent factors for PM. The nomogram was built based on these independent factors. The nomogram accuracy was evaluated by C-index and calibration curves. Bootstraps with 2,000 resamples were used to stabilize our results. A decision curve analysis (DCA) was utilized to evaluate the performance of our nomogram. The application and interpretation of this specific method are available online with a step-by-step tutorial [31, 34].
